# Wet season nitrogen export from a residential stormwater pond

**DOI:** 10.1371/journal.pone.0230908

**Published:** 2020-04-01

**Authors:** Jariani Jani, Mary G. Lusk, Yun-Ya Yang, Gurpal S. Toor

**Affiliations:** 1 Chemistry Department, University of Malaya, Kuala Lumpur, Malaysia; 2 Soil and Water Sciences Department, Gulf Coast Research and Education Center, University of Florida, Wimauma, Florida, United States of America; 3 Department of Environmental Science and Technology, University of Maryland, Maryland, United States of America; Trent University, CANADA

## Abstract

Stormwater runoff is recognized as a cause of water quality degradation because it may carry nitrogen (N) and other pollutants to aquatic ecosystems. Stormwater ponds are a stormwater control measure often used to manage stormwater runoff by holding a permanent pool of water, which reduces the peak flow, magnitude of runoff volume, and concentrations of nutrients and pollutants. We instrumented the outlet of a stormwater pond in an urban residential neighbourhood in Florida, United States to (1) investigate the concentration and composition of N forms during the summer rainy season (May to September 2016), and (2) determine the bioavailability of organic N in the stormwater pond with a bioassay experiment. A total of 144 outflow water samples over 13 storm events were collected at the outlet of the stormwater pond that collects runoff from the residential catchment. Samples were analysed for various inorganic N [ammonium (NH_4_–N), nitrate (NO_3_–N)], and organic N forms [dissolved organic nitrogen (DON), and particulate organic nitrogen (PON)]. Flow-weighted mean concentration of total N (TN) in pond outflow for all collected storm events was 1.3±1.42 mg L^-1^, with DON as the dominant form (78%), followed by PON and NO_3_–N (each at 8%), and NH_4_–N (6%). In the bioassay experiment, organic N (DON+PON) was significantly decreased by 25–28% after 5 days of incubation, suggesting that a portion of the DON carried from the pond outflow to receiving water bodies may be bioavailable. These results suggest that efforts to mitigate stormwater N outflows from urban ponds should incorporate both inorganic and organic N in management plans.

## Introduction

Stormwater runoff is recognized as an important source of nitrogen (N) in receiving water bodies, especially in urban landscapes with higher impervious surface area [1, 2]. The volume and flow velocity of stormwater runoff is generally higher on impervious surfaces than in natural areas. This increased water volume and velocity can change both the flow and quality of receiving streams, necessitating stormwater management practices that collect and mitigate stormwater flows [3–5]. Under the US Clean Water Act, states are required to monitor water quality, report water bodies that do not meet designated water quality criteria, and develop action plans to bring impaired water bodies back into compliance with water quality standards. One part of meeting this goal is to reduce contaminant transport to water bodies via stormwater runoff. As a result, best management practices (BMPs) or stormwater control measures (SCM) are designed to reduce, treat, or store polluted stormwater runoff [5].

Stormwater pond systems are the most widely used type of SCM throughout the United States [3, 5], with stormwater ponds use increasing globally as stormwater legislation addresses changes associated with urbanization [6]. In particular stormwater ponds have been found to number from 76,000 to nearly100,000 in Florida alone [7]. Stormwater ponds, also known as retention basins, wet ponds or wet detention basins are constructed to manage stormwater runoff by holding a permanent pool of water, thus reducing the peak runoff flow, tolerating the high magnitude of runoff volume, and potentially reducing the concentrations of pollutants and nutrients while often also contributing to groundwater recharge and river baseflow [5, 8, 9].

As compared to other SCMs, studies have shown that detention ponds are less effective in mitigating N loads [3, 10]. For example, a review of stormwater treatment options by Yang and Lusk [9] reports that N removal in ponds averages only 30–40%, whereas other SCMs such as bioretention and wetlands can achieve N removal up to 60–80%. A broad synthesis on stormwater BMPs by Koch et al. [4] reported that removal of N by stormwater ponds is variable, especially for inorganic N (NO_x_–N and NH_4_–N), with treatment efficacy ranging from up to 100% removal to no removal or even the net export of inorganic N from ponds. These ponds were reported to often be the source of NO_x_–N through biochemical transformations of sediment N that had settled in the ponds, in which in-pond mineralization of organic N was postulated as a cause of net export of inorganic N from the ponds [11]. Stormwater ponds may also increase the export of particulate organic matter and the N associated with particulates via internal production of algal biomass [12–14] Rosenzweig et al. [11] observed that the dissolved organic N (DON) load in pond outflow was two-times higher than the influent load, indicating the generation of DON in the ponds. Likewise, Lusk and Toor [15] and Williams et al. [16] provide evidence that urban stormwater ponds can have high levels of autochthonous dissolved organic matter production, suggesting that internal biogeochemical processes in the ponds could make the ponds net exporters of reactive N—potentially offsetting any pond water quality gains. Other recent studies have shown that DON can be a bioavailable N source in freshwater and marine ecosystems [17, 18]. This study investigates the bioavailability of DON in an urban stormwater pond as well as the concentration and composition of N in outflow from the pond during a summer rainy season. Further, our goal was to place DON outflow concentrations in context with other N forms in pond outflow and to assess the degree to which that DON may be bioavailable in downstream waters.

## Materials and methods

### Site description

**Fig 1:**
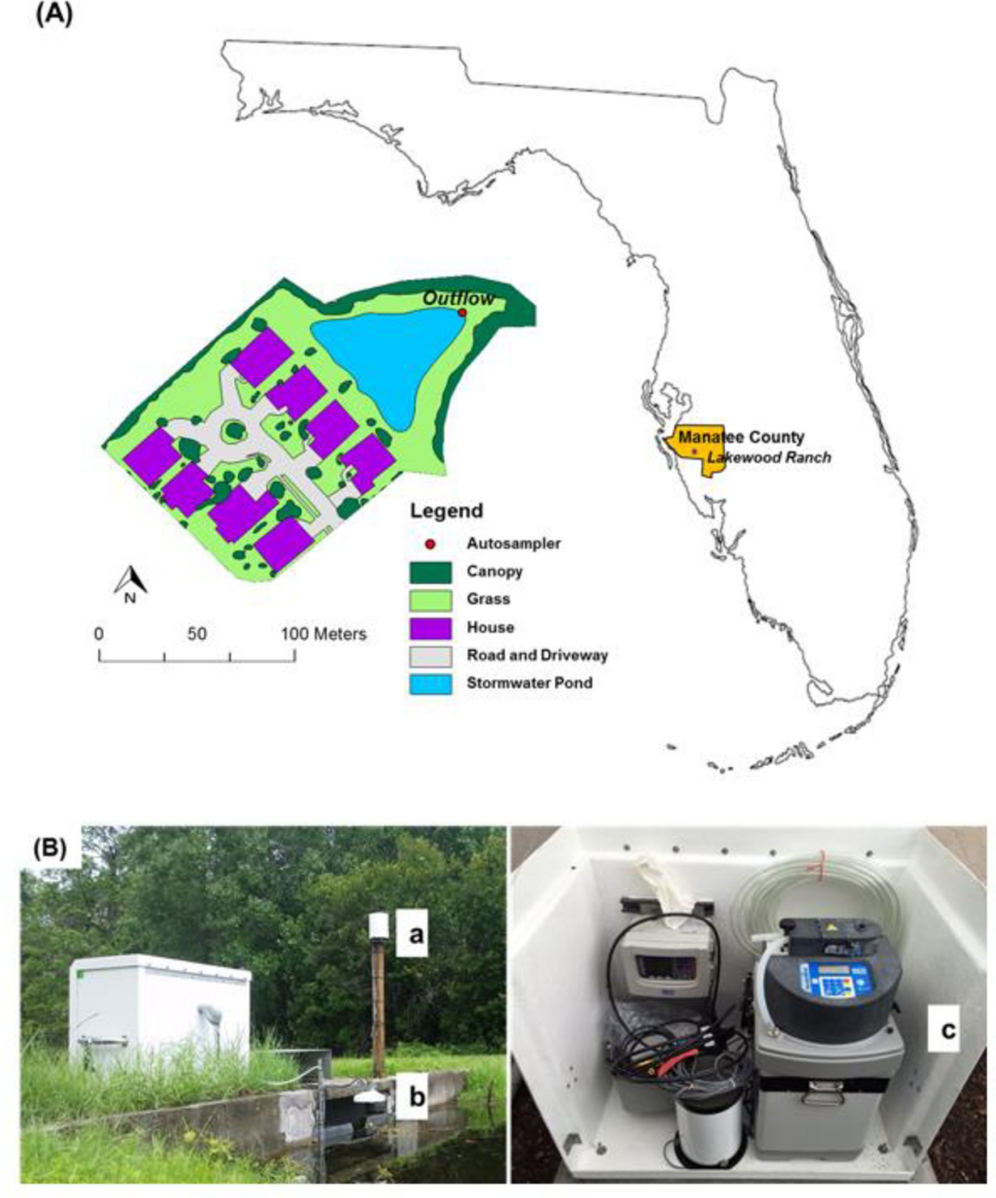
(A). Drainage area of the stormwater pond and (B) the various instruments installed including a) rain gauge, b) laser flow meter, and c) autosampler. (Photo courtesy of author).

The study was conducted at a stormwater wet pond located in the Lakewood Ranch residential neighborhood, Bradenton, Florida, United States. The stormwater pond has a permanent pool of 0.22 ha in size and was built in 2003, making the pond 13 years old at the time of this study. The soil within this catchment is predominantly Myakka fine sand series (Sandy, siliceous, hyperthermic Aeric Alaquods). The pond was designed to mimic and function as a natural water body that can retain water during storm events and filter constituents before they drain via piped conveyances into Braden River, a sub-watershed of the Tampa Bay estuary. The physical features of the catchment area for the pond are described in Table 1. The site is 2 ha with 37% impervious surface area (Fig 1). The study area is characterized by a sub-tropical climate with average annual rainfall of 143 cm, of which approximately 60% (86 cm) occurs during the summer rainy season months of June 1 through September 30.

**Table 1:** Area and percent land use of studied drainage area located in Lakewood Ranch, Bradenton, Florida, United States.

	Area (m^2^)	Percent (%)
**Pervious:**		63.0
**Canopy**	3539	17.5
**Grass**	9170	45.5
**Impervious:**		37.0
**Houses**	4581	22.7
**Roads**	2883	14.3
**Total**	20172	

### Bioassay experiments

We collected water samples from the pond and carried them through a 5-day incubation experiment to determine DON bioavailability. These water samples were collected on nine dates, spaced approximately 10 days apart during spring 2016. At each of the 9 collection dates, five 1-L grab samples were collected from the pond center and mixed in a bucket before taking a single composite 1-L sample in an amber glass bottle, which was kept on ice and transported to the lab for further processing and analyses. At the same time, three 1-L inoculum samples were collected in amber glass bottles at the most downstream site of Braden River, which receives a part of the pond outflow. Inoculum samples were filtered through 150 μm mesh to remove heterotrophic organisms prior to the bioassay experiment [19].

**Fig 2:**
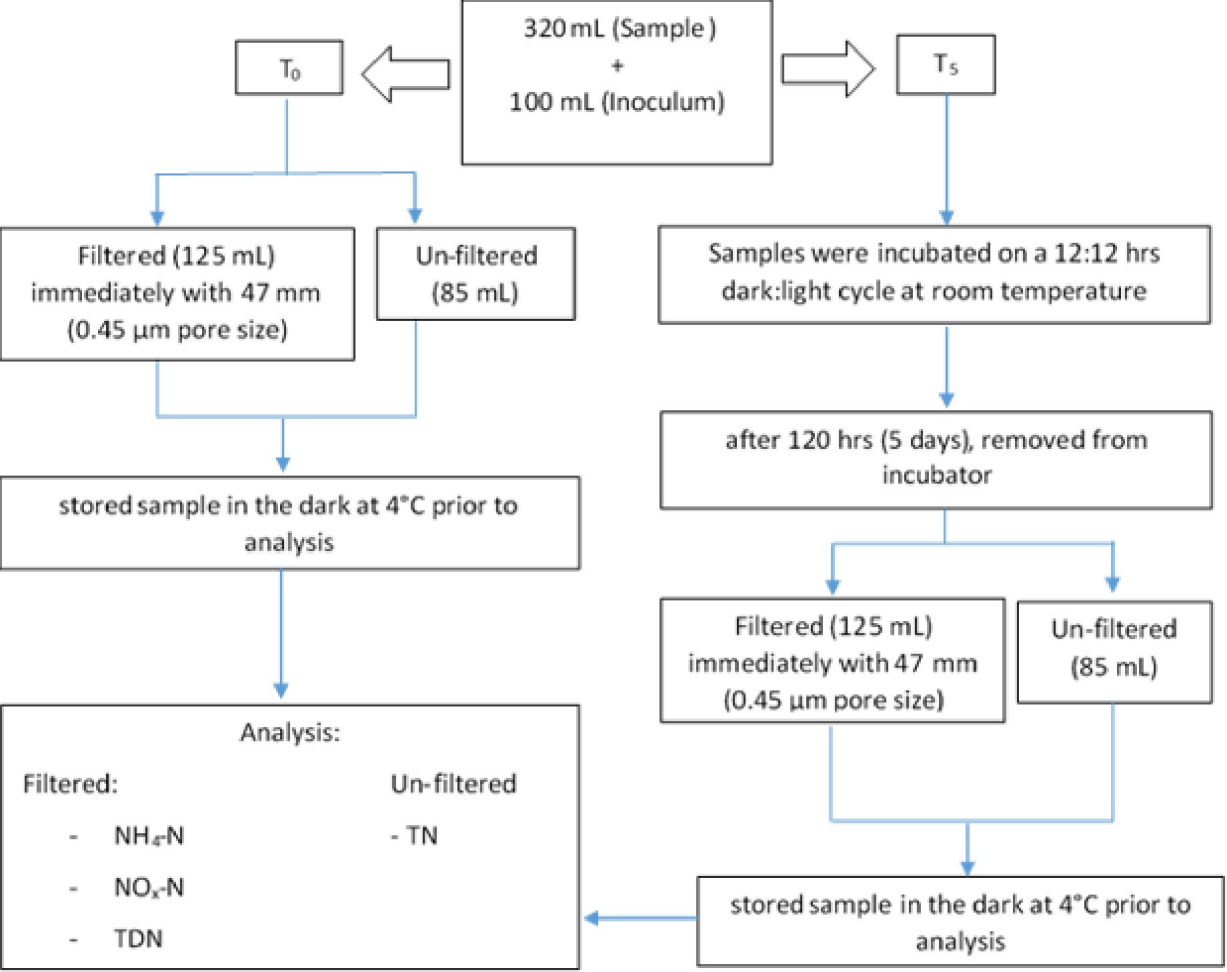
Flow chart showing various steps used in the bioassay study (Jariani and Toor, 2018) modified from (Osborne et al, 2013). T_0_ refers to initial (non-incubated) samples and T_5_ refers to samples designated for 5-days incubation.

The bioassay experiment was carried out following the methods described by Osborne et al. [15, 19–21], (Fig 2). In brief, 320 mL of pond water sample was mixed with 100 mL of filtered inoculum in 1-L amber glass bottle. The mixed samples were divided into two portions and designated as T_0_ (initial samples) and T_5_ (incubated samples). Subsamples of T_0_ were immediately filtered using Whatman #47 mm GF/F (0.45 μm pore size) filters and analyzed for total dissolved N (TDN), NH_4_–N and NO_x_–N, whereas the unfiltered samples were analyzed for TN (see Nitrogen Forms and Concentrations Analysis, below). The samples designated as T_5_ were incubated at a 12:12 hour dark: light cycle in the laboratory with temperature maintained at 22±2°C for 5 days, after which the samples were analyzed for TN, TDN, NH_4_–N, and NO_x_–N. After verifying normality of the T_0_ and T_5_ data, a paired t-test was used to test for statistical significance between the two sample points.

### Instrumentation

We have several years of collaboration with the Lakewood Ranch Homeowners’ Association (HOA) and we were granted permission for access to the community to conduct research. As this is a residential community managed by a HOA, no permits were required for our field work. At the pond outflow, an ultrasonic flow transmitter (Signature flowmeter, Teledyne Isco, NE) was installed to measure flow leaving the pond. A rain gauge (Teledyne ISCO, NE) was used to measure the rainfall and an autosampler (Avalanche, Teledyne ISCO, NE) was programmed to collect pond outflow samples according to pond flow characteristics (see Pond Outfall Sampling, below). The autosampler had 14 (950 ml) plastic sample collection bottles, with a built-in refrigerator to keep the sample at 4°C. The rain gauge and autosampler were programmed to download the data into FlowLink 5.1 software (Teledyne ISCO, NE), which was used to track rainfall depth, pond outflow volume and sample collection time. Sample dates and associated rainfall depths and pond outflow volumes are presented in Table 2.

**Table 2:** Number of samples collected, rainfall amount (cm), and pond outflow volume (m^3^) for 13 storm events from May to September 2016.

Date	Event	No. of Collected Samples	Total Rainfall (cm)	Outflow Volume (m^3^)
**05/04/16**	1	14	1.68	6.9
**05/07/16**	2	7	0.05	14.37
**06/09/16**	3	12	12.75	879.12
**06/12/16**	4	2	0.05	25.20
**08/08/16**	5	11	3.30	18.27
**09/08/16**	6	14	2.49	955.08
**08/10/16**	7	6	5.38	479.64
**08/13/16**	8	8	0.36	124.80
**08/20/16**	9	14	1.40	466.98
**08/31/16**	10	14	5.82	210.24
**09/01/16**	11	14	0.23	415.44
**09/02/16**	12	9	2.97	69.36
**09/12/16**	13	14	0.03	64.32

### Pond outflow sampling

Samples were collected in spring and summer 2016 from the pond outflow. At the beginning of the collection period, as the water level in the outflow pipe was low (~0.4 cm) because of the preceding dry season, the flowmeter was programmed to enable sampling when the rain gauge captured 0.25 cm of rain and the level of water in the outflow pipe was 0.65 cm for the duration of 5 minutes. As the rainy season progressed, the level of water increased and there was outflow from the pond at all times, therefore, the sampling program was changed (e.g., level of water to start sampling and sampling intervals). The threshold level to initiate sampling ranged from 0.65 to 20.32 cm in the outflow pipe, and the sampling intervals between each sample varied between 10 min to 6 hr intervals. Samples were taken to the laboratory within 24 hr of collection. A total of 13 storm events (144 samples) were collected. Prior to the sampling, all sampling containers used were acid washed (10% hydrochloric acid, HCl) and rinsed with deionized water.

### Nitrogen forms and concentrations analysis

Samples for the bioassay experiment and from the pond outflow collections were analyzed using a continuous flow ion analyzer (AA3, Seal Analytical Inc., Mequon, WI) for NH_4_–N and NO_x_–N (NO_3_–N + NO_2_–N) with USEPA Method 350.1 and 353.2, respectively. The filtered and unfiltered samples were analyzed for TDN and TN, respectively, using oxidative combustion-chemiluminescence (ASTM, 2015) by a Total Organic Carbon Analyzer (TOC-L CPH/CPN, Shimadzu Corp., Columbia, MD). Other N forms were calculated as follows: particulate organic nitrogen (PON) = TN–TDN and DON = TDN–(NO_x_–N + NH_4_–N).

### Flow-weighted mean concentrations analysis

Flow-weighted mean concentrations (FWMC) (mg L^-1^) were used to quantify the concentration proportional to a corresponding flow volume. The FWMC for each parameter were derived from the concentrations, sample time window, and flow volume for each sample. The equation is as follows:

$$FWMC=\frac{\sum_{1}^{n}\left(ci*ti*qi\right)}{\sum_{1}^{n}\left(ti*qi\right)}$$ (1)

where *ci* = concentration in i^th^ sample

*ti* = time (min) window of i^th^ sample

*qi* = flow volume in i^th^ sample

This equation allows the concentration in each sample to be considered in light of the time window and flow volume associated with it [22, 23].

## Results and discussion

### Bioavailability of organic nitrogen

**Fig 3:**
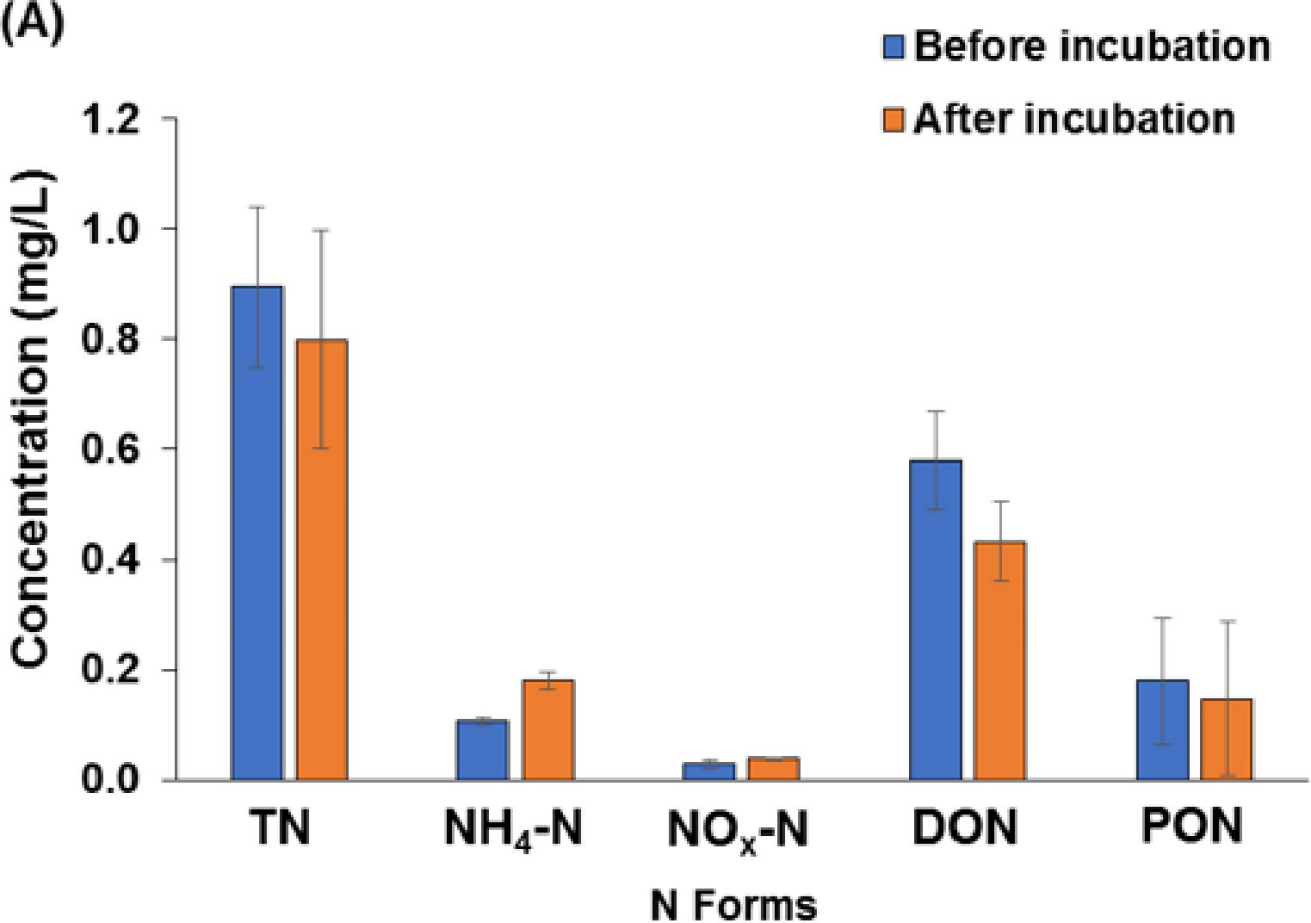
Concentration of nitrogen forms before and after the 5-day bioassay experiment.

In the bioassay experiment, organic N (DON+PON) was significantly decreased (p<0.05) after 5-days of incubation. Mean DON concentrations were reduced by 25% (range: 18 to 30%), suggesting that a portion of DON was bioavailable (Fig 3). The mean PON concentration also showed a 28% (range: 2 to 52%) reduction during the incubation (Fig 3), suggesting that a portion of PON was also subject to biogeochemical transformation. In contrast to organic N, NO_x_–N concentrations increased by 35–39% (mean: 30±38%) and NH_4_–N concentrations increased by 9–39% (mean: 39±16%) (Fig 3). This finding is similar to Seitzinger and Sanders [24] and Jani and Toor [21], who reported an increase in DIN concentrations and decrease in DON concentrations in the bioassay experiments. This net increase in inorganic N was likely due to the mineralization of organic N to NH_4_–N, which was further nitrified to NO_x_–N [25–27]. However, it should be noted that our bioassay experiment likely took place with elevated oxygen conditions relative to what would be expected in the ponds *in situ*. If the ponds were anoxic or stratified, we might not observe nitrification rates as high as reported by our laboratory bioassay study. Indeed if this was the case (low oxygen and stratified pond environment, then in situ pond nitrification may be limited [3, 28].

Decomposition of PON by microbial action might explain why DON was the most abundant N form in our stormwater pond (Fig 3) and why several authors have noted that these urban stormwater treatment ponds are net DON exporters (e.g., Lusk and Toor [15]). Lusk and Toor [15] study did not include analysis of PON but did show that DON concentration in pond outflow (mean 1.85 mg L^-1^) was higher than that in pond inflow (mean 1.09 mg/L), suggesting that in-pond processes, including decomposition of PON, generated new DON. Further, Lusk and Toor [15] showed through high resolution mass spectrometry that the chemical composition of the DON was transformed inside the pond to produce DON compounds that were lower in molecular weight and more aromatic than those in the incoming runoff—again suggesting a degree on in-pond processing that changes the N signal of urban runoff. Numerous studies have reported that microbial populations are both consumers and producers of DON because they use and release reactive N exudates that can be consumed and reformed [29, 30]. Our study is the first of which we are aware that includes inspection of both DON and PON biodegradability within an urban stormwater pond, and that a third to a half of these ON forms were bioavailable in our study indicates that ON can be dynamic in urban aquatic systems. By including PON in our study, we show that a portion of PON can be utilized and transformed by microbes in the pond, implying that physical processes like sedimentation of particulates may have limited efficiency for reducing N export from the pond if PON degradation and utilization result in the generation of DON and/or dissolved inorganic N. In this way, the pond would function less as an N remover and more as merely an N transformer, though we did not specifically look at this process and it is certainly one that warrants further study [15, 16].

One important implication of this work comes from the fact that wet stormwater ponds are often connected to receiving water bodies and bioavailable N that leaves these ponds can thus add to the reactive N loads of downstream waters. We recognize that reactive N would likely be transported from urban landscapes to receiving streams even if stormwater ponds were not present, but our data highlights how outflows from these ponds should be recognized as potentially carrying a reactive N load. Gold et al. [3] observed little to no improvement in downstream water quality and nutrient concentrations after watershed-scale implementation of wet stormwater ponds in an urbanizing coastal area. A growing number of researchers are recognizing that in-pond nutrient cycling may make urban stormwater ponds function as sources of dissolved nutrients [31, 32], and there is a need for new research that investigates the mechanistic controls on nutrient cycling and downstream fate in these urban features of the built environment. We provide evidence here of the dynamic nature of in-pond DON and PON, and future studies should investigate this across temporal and spatial scales and in environments with varying ON sources such as with different types of vegetation.

### Distribution of nitrogen forms in pond outflow waters

**Fig 4:**
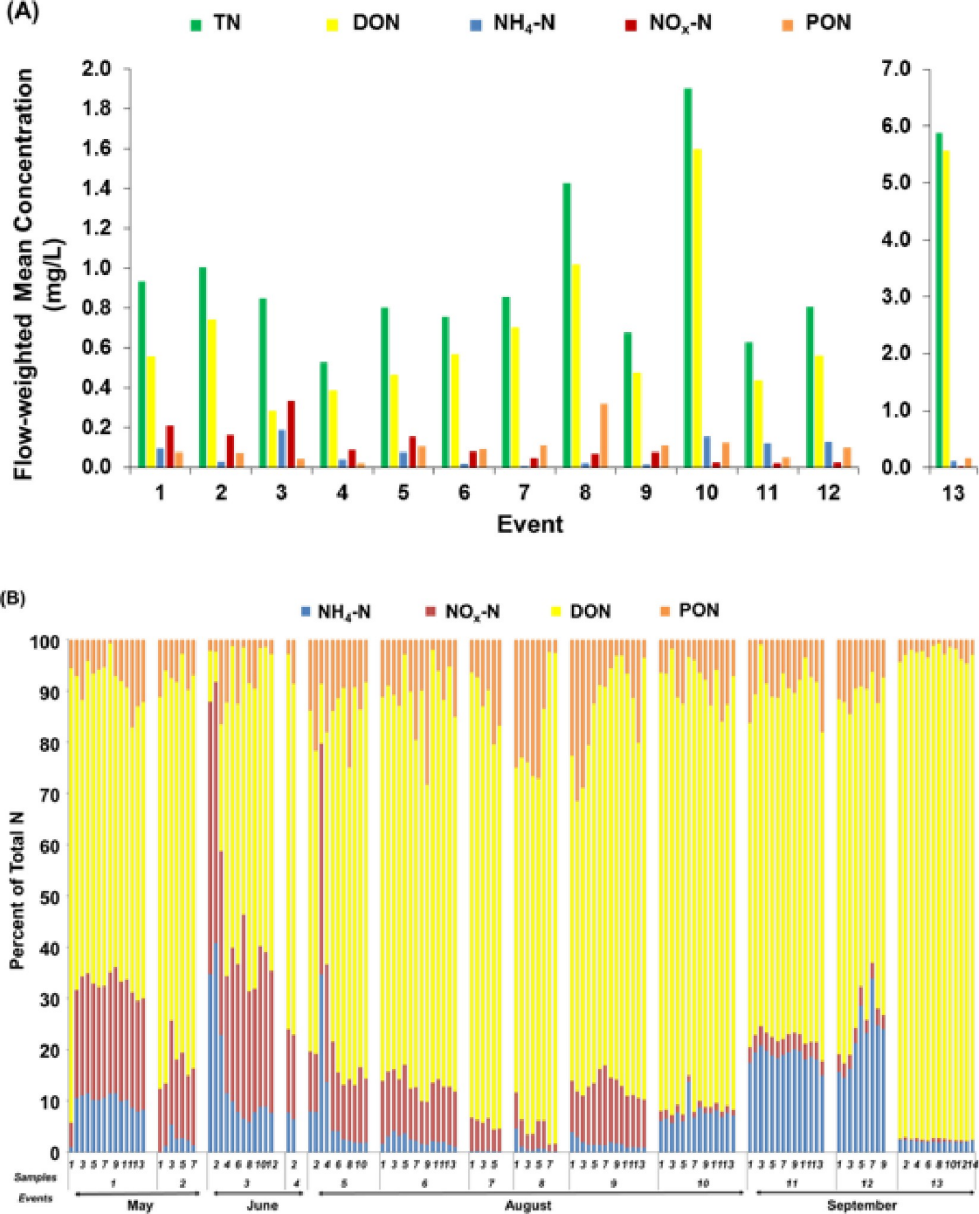
(A) Flow-weighted mean concentration (FWMC), and (B) composition of nitrogen forms in individual pond outflow samples from May to September 2016 (n = 13 storms; n = 144 samples).

Given the above result that a portion of the ON in our pond was bioavailable, our second objective was to place ON concentrations and proportions in context with inorganic N forms in the pond outflow. The FWMC of pond outflow TN for all storm event was 1.31±1.42 mg L^-1^ (range: 0.53 to 5.88 mg L^-1^). For the entire wet season, DON concentration was higher than other N forms for most storm events and ranged from 0.28 to 5.56 mg L^-1^ (mean: 1.03 mg L^-1^), followed by PON (0.02 to 0.32 mg L^-1^; mean: 0.11 mg L^-1^), NO_x_–N (0.02 to 0.33 mg L^-1^; mean: 0.10 mg L^-1^), and NH_4_–N (0.001 to 0.19 mg L^-1^; mean: 0.07 mg L^-1^) (Fig 4). The highest mean concentration of TN for the season was observed in the last event with 5.88 mg L^-1^ (Fig 4).

There are scarce reports in the literature that include both DON and PON in investigations of N forms in urban runoff and stormwater ponds. Our observation that DON was the dominant N form in the pond outflow was similar to findings by Lusk and Toor [15], who reported DON as the dominant form of N in outflow from a residential wet pond, and in line with Taylor et al. [33] who observed that DON was likewise the dominant N form in urban runoff. At the beginning of the season, (events 1 to 5), the ranking of strength in distribution among N forms in the pond outflow was DON > NO_x_–N > NH_4_–N > PON (Fig 4). This pattern remained until the middle of the season (events 6 to 9) when PON increased as the second highest N form after DON, suggesting at this stage of the season, the stormwater pond may be highly influenced by sources of particulate N, such as grass clippings, leaf litter, and plant debris that was carried by stormwater runoff and that did not settle in the pond (Fig 4). A study by Rosenzweig et al. [11] reported that particulate N ranged from 26 to 38% of total N transport and the fraction was greater during storm events. At the end of the season in September (events 10 to 13), our data showed that NH_4_–N concentrations increased, but DON remained as having the highest concentration, especially in event 13 that resulted an anomalous one-time spike in TN (Fig 4), the source of which we did not investigate.

Our data revealed that organic N was a significant portion of N in the pond outflow, suggesting that reduction of NO_x_–N and NH_4_–N alone might not be adequate to mitigate downstream loading of TN. For example, in our study area, summer rainy season bans on turf fertilizers that contain inorganic N are common. The premise behind these bans is that summer rains drive inorganic N to runoff and stormwater ponds. Our study demonstrates that reactive N may still be transported from these ponds even when such bans are in place. Furthermore, the FWMC and proportion of DON remained fairly steady throughout the wet season, suggesting a constant catchment and/or in-pond source of DON. Potential catchment sources of ON to urban runoff are numerous and include atmospheric deposition, plant material, soils, fertilizers, reclaimed water used for irrigation, and pet waste [9, 34–36].

Overall, the composition of N forms in our pond outflow are similar with our previous study [17] conducted in a longitudinal gradient from freshwater (Braden River) to estuarine (Tampa Bay) systems that showed organic N as the dominant N form (86% TN). The Braden River is directly connected to our pond outflow, together with many other ponds in the Braden river watershed, suggesting that pond outflow from residential areas is one contributor of organic N input to the river ecosystems.

## Conclusions

This study was conducted to investigate the magnitude of organic and inorganic N forms and the bioavailability of N in a stormwater pond during a rainy season. The main conclusions from the study are (1) pond outflow was dominated by DON (78% of TN over the season), and (2) a portion of DON in stormwater ponds was bioavailable, ranging from 18–30% of the DON pool and 2–52% of the PON pool. These suggest that stormwater pond management for N removal should incorporate not only inorganic N but also organic N strategies. From an ecosystem standpoint, we also argue that greater emphasis should be placed on the role of urban wet stormwater ponds in downstream ecology. Our evidence of ON bioavailability suggests that these ponds may transport reactive ON and thus exacerbate water quality degradation in downstream waters.

## Supporting information

S1 Data (XLSX)

## References

[1] Li L, Davis AP. Urban stormwater runoff nitrogen composition and fate in bioretention systems. Environmental science & technology. 2014. 10.1021/es4055302 24571092

[2] Nagy CR, Lockaby GB, Kalin L, Anderson C. Effects of urbanization on stream hydrology and water quality: the Florida Gulf Coast. Hydrological Processes. 2012;26(13):2019–30.

[3] Gold A, Thompson S, Piehler M. Water quality before and after watershed-scale implementation of stormwater wet ponds in the coastal plain. Ecological Engineering. 2017;105:240–51.

[4] Koch BJ, Febria CM, Gevrey M, Wainger LA. Nitrogen removal by stormwater management structures: a data synthesis. Journal of the American Water Resources Association. 2014;50(6):1594–607.

[5] Collins K, Lawrence T, Stander E, Jontos R, Kaushal S, Newcomer T, et al Opportunities and challenges for managing nitrogen in urban stormwater: A review and synthesis. Ecological Engineering. 2010;36(11):1507–19. 10.1016/j.ecoleng.2010.03.015 WOS:000283010600002.

[6] Betts AT, Alsharif KA. Assessment of a countywide stormwater pond improvement program. Urban Water Journal. 2014;11(1):11–9.

[7] Sinclair JS, Reisinger AJ, Bean E, Adams CR, Reisinger LS, Iannone BV III. Stormwater ponds: An overlooked but plentiful urban designer ecosystem provides invasive plant habitat in a subtropical region (Florida, USA). Science of The Total Environment. 2020;711:135133 10.1016/j.scitotenv.2019.135133 31837878

[8] Hancock GS, Holley JW, Chambers RM. A Field‐Based Evaluation of Wet Retention Ponds: How Effective Are Ponds at Water Quantity Control? 1. JAWRA Journal of the American Water Resources Association. 2010;46(6):1145–58.

[9] Yang Y-Y, Lusk MG. Nutrients in Urban Stormwater Runoff: Current State of the Science and Potential Mitigation Options. Current Pollution Reports. 2018;4(2):112–27. 10.1007/s40726-018-0087-7

[10] Harper HH, Baker DM. Evaluation of current stormwater design criteria within the state of Florida. Orlando, Florida: Environmental Research & Design, Inc; 2007.

[11] Rosenzweig BR, Smith JA, Baeck ML, Jaffe PR. Monitoring Nitrogen Loading and Retention in an urban strormwater Detention Pond. Journal of Enviromental Quality. 2011;40:598–609. 10.2134/jeq2010.0300 21520767

[12] Gold AC, Thompson SP, Piehler MF. The Effects of Urbanization and Retention‐Based Stormwater Management on Coastal Plain Stream Nutrient Export. Water Resour Res. 2019;55(8):7027–46.

[13] DeLorenzo ME, Thompson B, Cooper E, Moore J, Fulton MH. A long-term monitoring study of chlorophyll, microbial contaminants, and pesticides in a coastal residential stormwater pond and its adjacent tidal creek. Environmental Monitoring and Assessment. 2012;184(1):343–59. 10.1007/s10661-011-1972-3 21409361

[14] Lewitus AJ, Brock LM, Burke MK, DeMattio KA, Wilde SB. Lagoonal stormwater detention ponds as promoters of harmful algal blooms and eutrophication along the South Carolina coast. Harmful Algae. 2008;8(1):60–5.

[15] Lusk M, Toor G. Biodegradability and Molecular Composition of Dissolved Organic Nitrogen in Urban Stormwater Runoff and Outflow Water from a Stormwater Retention Pond. Environmental Science & Technology. 2016;50(7):3391–8. 10.1021/acs.est.5b05714 WOS:000373655800012. 26967971

[16] Williams CJ, Frost PC, Xenopoulos MA. Beyond best management practices: pelagic biogeochemical dynamics in urban stormwater ponds. Ecological Applications. 2013;23(6):1384–95. 10.1890/12-0825.1 24147410

[17] Petrone KC, Richards JS, Grierson PF. Bioavailability and composition of dissolved organic carbon and nitrogen in a near coastal catchment of soth- western Australia. Biogeochemistry. 2009;92:27–40.

[18] Bronk D, See J, Bradley P, Killberg L. DON as a source of bioavailable nitrogen for phytoplankton. Biogeosciences. 2007;4(3):283–96.

[19] Osborne D, Podgorski D, Bronk D, Roberts Q, Sipler R, Austin D, et al Molecular-level characterization of reactive and refractory dissolved natural organic nitrogen compounds by atmospheric pressure photoionization coupled to Fourier transform ion cyclotron resonance mass spectrometry. Rapid Communications in Mass Spectrometry. 2013;27(8):851–8. 10.1002/rcm.6521 WOS:000316624400001. 23495054

[20] Lusk M, Toor G. Dissolved organic nitrogen in urban streams: Biodegradability and molecular composition studies. Water Research. 2016;96:225–35. 10.1016/j.watres.2016.03.060 WOS:000376211700023. 27058880

[21] Jani J, Toor GS. Composition, sources, and bioavailability of nitrogen in a longitudinal gradient from freshwater to estuarine waters. Water Research. 2018;137:344–54. 10.1016/j.watres.2018.02.042 29571112

[22] Charbeneau RJ, Barrett ME. Evaluation of methods for estimating stormwater pollutant loads. Water Environment Research. 1998;70(7):1295–302.

[23] Sansalone JJ, Buchberger SG. Partitioning and first flush of metals in urban roadway storm water. Journal of Environmental engineering. 1997;123(2):134–43.

[24] Seitzinger SP, Sanders R, Styles R. Bioavailability of DON from natural and anthropogenic sources to estuarine plankton. Limnology and Oceanography. 2002;47(2):353–66.

[25] Bijoor NS, Czimczik CI, Pataki DE, Billings SA. Effects of temperature and fertilization on nitrogen cycling and community composition of an urban lawn. Global Change Biology. 2008;14(9):2119–31.

[26] Butler SM, Melillo JM, Johnson J, Mohan J, Steudler PA, Lux H, et al Soil warming alters nitrogen cycling in a New England forest: implications for ecosystem function and structure. Oecologia. 2012;168(3):819–28. 10.1007/s00442-011-2133-7 21983640 PMC3277705

[27] Causse J, BaurèS E, Mery Y, Jung A-V, Thomas O. Variability of N export in water: a review. Critical Reviews in Environmental Science and Technology. 2015;45(20):2245–81.

[28] Song K, Xenopoulos MA, Buttle JM, Marsalek J, Wagner ND, Pick FR, et al Thermal stratification patterns in urban ponds and their relationships with vertical nutrient gradients. Journal of environmental management. 2013;127:317–23. 10.1016/j.jenvman.2013.05.052 23810965

[29] Fasching C, Behounek B, Singer GA, Battin TJ. Microbial degradation of terrigenous dissolved organic matter and potential consequences for carbon cycling in brown-water streams. Scientific reports. 2014;4:4981 10.1038/srep04981 24828296 PMC4021337

[30] Guillemette F, del Giorgio PA. Simultaneous consumption and production of fluorescent dissolved organic matter by lake bacterioplankton. Environmental microbiology. 2012;14(6):1432–43. 10.1111/j.1462-2920.2012.02728.x 22429445

[31] Gold AC, Thompson SP, Piehler MF. Nitrogen cycling processes within stormwater control measures: A review and call for research. Water research. 2018. 10.1016/j.watres.2018.10.036 30513447

[32] Beckingham B, Callahan T, Vulava VM. Stormwater ponds in the southeastern US coastal plain: hydrogeology, contaminant fate, and the need for a social-ecological framework. Frontiers in Environmental Science. 2019;7:117.

[33] Taylor GD, Fletcher TD, Wong TH, Breen PF, Duncan HP. Nitrogen composition in urban runoff—implications for stormwater management. Water Research. 2005;39(10):1982–9. 10.1016/j.watres.2005.03.022 15921721

[34] Lusk MG, Toor G. S., and Inglett P. W. Organic nitrogen in residential stormwater runoff: Implications for stormwater management in urban watersheds. Science of the Total Environment. 2019 10.1016/j.scitotenv.2019.135962 31863977

[35] Cornell S. Atmospheric nitrogen deposition: revisiting the question of the invisible organic fraction. Procedia Environmental Sciences. 2011;6:96–103. 10.1016/j.envpol.2010.11.014 21131113

[36] Toor GS, Lusk M. Reclaimed water use in the landscape: constituents of concern in reclaimed water. Gainesville (Florida): University of Florida, Institute of Food and Agricultural Sciences, Florida Cooperative Extension Service 2011.

